# Targeting the Cav1.2-KCa1.1 axis prevents rabies virus internalization and protects against lethal infection

**DOI:** 10.1371/journal.ppat.1014675

**Published:** 2026-10-06

**Authors:** Ziruo Sun, Jinqiu Wang, Zhiyuan Wen, Lei Shuai, Wenjing Sun, Mengjie Yang, Jinyu Wang, Junyu Chen, Tengteng Mi, Xianjing Zhou, Peiran Zhou, Nannan Lv, Zhenbo Zhao, Jinying Ge, Weiye Chen, Xijun Wang, Chong Wang, Zhigao Bu, Jinliang Wang

**Affiliations:** 1 State Key Laboratory of Animal Disease Control and Prevention, Harbin Veterinary Research Institute, Chinese Academy of Agricultural Sciences, Harbin, People’s Republic of China; 2 National High Containment Laboratory for Animal Disease Control and Prevention, Harbin, People’s Republic of China; 3 Jiangsu Co-innovation Center for Prevention and Control of Important Animal Infectious Diseases and Zoonoses, Yangzhou University, Yangzhou, People’s Republic of China; Université Paris-Saclay: Universite Paris-Saclay, FRANCE

## Abstract

Rabies is an almost always fatal disease caused by rabies virus (RABV). Once RABV enters the central nervous system, the mortality rate is almost 100%. Therefore, preventing RABV from entering the nervous system is crucial for the development of anti-RABV therapeutics. Here, we found that the L-type calcium channel Cav1.2 pore-forming subunit (Ca_v_1.2) and potassium calcium-activated channel subfamily M alpha 1 (KCa1.1) are involved in the internalization of RABV using an siRNA approach. RABV glycoprotein interacts with Ca_v_1.2, a process that is required for the subsequent activation of the channel. The activated Ca_v_1.2 then interacts with and activates KCa1.1 to regulate the endocytosis of RABV by promoting the F-actin polymerization. Importantly, diltiazem, a blocker of Ca_v_1.2 and an FDA-approved drug, has been shown to substantially reduce mortality in mice infected with RABV. Approximately 70% of mice survived following treatment with diltiazem at 50 mg/kg, whereas the 25 mg/kg dose resulted in 26% survival. Our findings suggest that Ca_v_1.2 is a promising target for the development of antiviral drugs against rabies and enhances our comprehension of the RABV entry mechanism.

## Author summary

Rabies is a zoonotic infectious disease that is almost invariably fatal once clinical symptoms appear, causing approximately 59,000 deaths annually. While effective post-exposure prophylaxis exists, the expensive cost and chronic global shortage of rabies immunoglobulins leave underserved populations—particularly in rural areas of Asia and Africa—dangerously unprotected. Therefore, discovering affordable, readily accessible therapeutic alternatives to block early viral infection is an urgent global public health need. In this study, we identified that the rabies virus hijacks Ca_v_1.2-KCa1.1 axis to enter cells. By targeting this newly identified axis in the viral entry process, we repurposed diltiazem, a widely used, low-cost, and FDA-approved cardiovascular drug that blocks the Ca_v_1.2 channel. Remarkably, intramuscular administration of diltiazem (50 mg/kg) successfully rescued approximately 70% of mice challenged with a lethal dose of rabies virus. Our findings suggest that repurposed diltiazem shows potential as an early intervention strategy to limit RABV infection. By identifying a safe, mass-produced clinical drug as a potent viral entry inhibitor, this work provides a scalable intervention that could complement existing vaccines and rabies immunoglobulins. This strategy has profound potential to mitigate healthcare inequities in resource-limited regions and significantly advance global efforts to eliminate rabies-related deaths.

## Introduction

Rabies, a zoonotic disease caused by the rabies virus (RABV), is almost invariably fatal once the virus reaches the central nervous system (CNS) and clinical symptoms develop [1, 2]. Despite vaccines and therapeutic antibodies against RABV are available, rabies remains a significant health threat, particularly in underdeveloped regions where access to medical resources and vaccination programs is limited [3]. Globally, the disease is responsible for approximately 59,000 fatalities annually, with a disproportionate impact on children under the age of 15, accounting for nearly half of the cases [4–6]. This highlights an urgent need for the design of cost-effective antiviral drugs capable of preventing mortality associated with rabies infection.

RABV, a member of the Lyssavirus genus within the Rhabdoviridae family, possesses a single-stranded, negative-sense RNA genome that encodes five essential structural proteins: nucleoprotein, phosphoprotein, matrix protein, glycoprotein (G), and RNA-dependent RNA polymerase [7]. The G protein is responsible for binding to specific receptors and then mediating viral internalization through clathrin-mediated endocytosis with the help of actin [8]. Current studies have identified five potentially RABV receptors, including acetylcholine receptor subunit alpha [9], neural cell adhesion molecule [10], low-affinity nerve-growth factor receptor [11], metabotropic glutamate receptor 2 [12] and neuropilin 2 [13]. Additionally, transferrin receptor 1 [14], AP2-associated kinase 1 [15], and integrins [16] have also been implicated in the entry process of RABV, indicating a complex interaction of multiple membrane proteins during RABV entry. Since the mortality rate approaches 100% once the RABV has invaded the CNS [17], understanding the underlying mechanism of RABV entry and identifying key drug targets involved in viral entry are crucial for the development of antiviral drugs to block early infection.

Ion channels are present on the cell membrane and can modulate many cellular functions, such as nerve and muscle activity, the heartbeat and blood pressure [18]. Given their importance, it is well-established that ion channels are targeted by a wide array of therapeutic agents for various diseases [19]. Previous studies have revealed that ion channels, particularly calcium (Ca^2+^) channels and potassium (K^+^) channels, are involved in the infection of numerous viruses [18, 20]. For instance, two-pore calcium channels control Ebola virus entry [21]; L-type calcium channel Ca_v_1.2 pore-forming subunit (Ca_v_1.2) is required for the entry of SARS-CoV-2 and influenza virus [22, 23]; voltage-gated K^+^ channels affect the infection of Merkel cell polyomavirus and Simian virus 40 [24]; twin-pore domain K^+^ channels are required for the early infection of Bunyamwera virus and Hazara virus [25]. Recent studies have demonstrated that potassium calcium-activated channel subfamily M alpha 1 (KCa1.1) plays a critical role in influenza virus entry by regulating clathrin-mediated endocytosis [26, 27]. However, whether calcium channels and K^+^ channels are important for RABV infection remains unclear.

In this study, we found that Ca_v_1.2 facilitates the internalization of RABV through activating KCa1.1. The FDA-approved drug diltiazem [28], a known inhibitor of Ca_v_1.2 [29], decreases RABV infection *in vitro* and *in vivo*. Mice infected with a lethal dose of RABV had a survival rate of approximately 70% after diltiazem treatment. Our findings suggest that diltiazem may serve as a potential therapeutic agent for RABV infection, and that Ca_v_1.2 and KCa1.1 are promising targets for the development of antiviral drugs against rabies.

## Results

### KCa1.1 is required for RABV infection

In our previous study, we performed a genome-wide siRNA screen in HEK293 cells to identify host factors required for RABV infection, using 64,755 siRNAs targeting 21,585 human genes. This screen revealed 16 genes related to potassium channels, with downregulation of KCNMA1 showing the greatest impact on RABV replication (Fig 1A and S1 Table). KCNMA1 encodes the pore-forming subunit of the large conductance voltage- and Ca^2+^-activated K^+^ channel (KCa1.1), which is a seven-transmembrane protein, and paxilline is a known functional antagonist of KCa1.1 [30].To confirm this observation, we knocked down KCa1.1 expression in N2a cells and HEK293 cells using specific siRNA targeting KCa1.1 mRNA, respectively. Quantitative RT-PCR (qPCR) analysis confirmed that compared to cells transfected with scrambled siRNA, KCa1.1 mRNA levels were significantly reduced in both siKCa1.1-transfected cells at 48 h post-transfection (Fig 1B). At 72 h post-transfection, N2a cells or HEK293 cells were infected with ERA-EGFP (MOI 0.01), a recombinant RABV ERA strain that expresses enhanced green fluorescent protein (ERA-EGFP), and the virus released into the culture supernatants was quantified by viral titration at 24 h and 48 h post-infection (p.i.). We found that the viral titers were significantly decreased in both KCa1.1-silenced cells (Fig 1C and 1D), which confirms our screen result.

**Fig 1:**
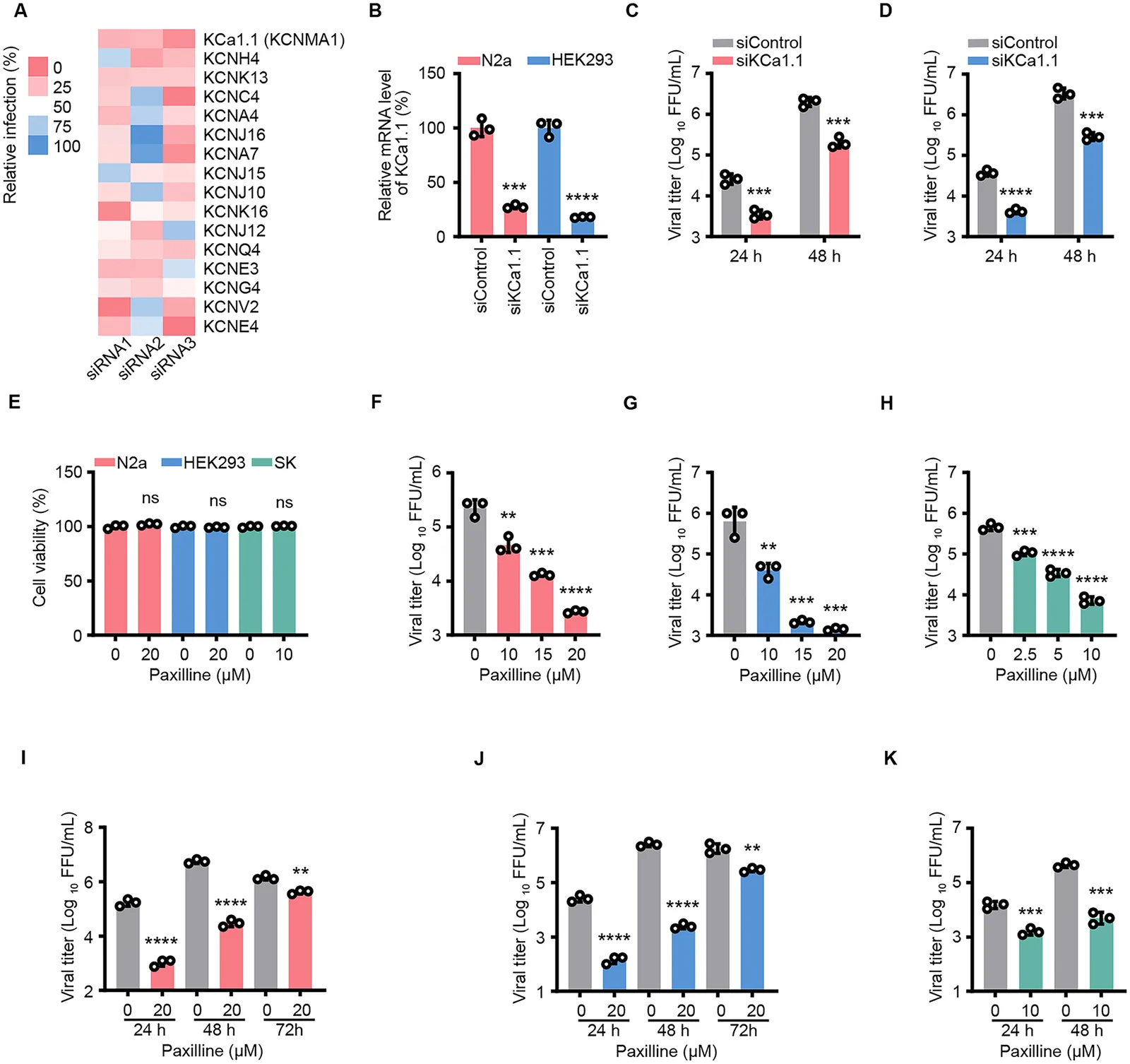
KCa1.1 is required for RABV infection. **(A)** High-throughput siRNA screening identified KCa1.1 as a critical host factor for RABV infection, showing the most significant impact among potassium channel-related genes. **(B)** KCa1.1 knockdown significantly reduced KCa1.1 mRNA levels in N2a and HEK293 cells. **(C and D)** KCa1.1 knockdown significantly reduced RABV replication at 24 h and 48 h post-infection in N2a and HEK293 cells. **(E)** Paxilline treatment did not affect the viability of N2a, HEK293, and SK cells. **(F–H)** Paxilline treatment significantly inhibited RABV replication in a dose-dependent manner in N2a, HEK293, and SK cells. **(I and J)** Paxilline treatment reduced RABV replication at 24 h, 48 h, and 72 h post-infection in N2a and HEK293 cells. **(K)** Paxilline treatment reduced RABV replication at 24 h and 48 h post-infection in SK cells. Data shown in B–K are presented as means  ±  s.d. (n  =  3 biologically independent experiments). Statistical analysis was performed using unpaired, two-tailed Student’s t-test. ns, not significant. * P  <  0.05; ** P  <  0.01; *** P  <  0.001; **** P  <  0.0001.

To further confirm this effect of KCa1.1 on RABV infection, we performed dose-response assays with paxilline in N2a, HEK293, and human neuroblastoma SK-N-SH cells (SK cells). Paxilline is a selective, state-dependent inhibitor of KCa1.1 that binds to the inner pore region of the channel and inhibits K^+^ conductance, thereby preventing KCa1.1 activation [30, 31]. Cells were pre-treated with various concentrations of paxilline for 1 h prior to infection with ERA-EGFP (MOI 0.01). The viral titers in the supernatants were titrated at 48 h p.i.. We found that paxilline treatment had no effect on cell viability in any of the three cell lines, but significantly inhibited the replication of RABV in a dose-dependent manner (Fig 1E-1H). Furthermore, we performed a time-course assay using a fixed concentration of paxilline. N2a or HEK293 cells were pre-treated with paxilline at a concentration of 20 μM, and viral titers in the supernatants were determined at 24 h, 48 h, and 72 h p.i., whereas SK cells were pre-treated with 10 μM paxilline and viral titers were determined at 24 h and 48 h p.i. The results showed that paxilline treatment inhibited RABV infection at the corresponding tested time points (Fig 1I-1K). Collectively, these results demonstrate that activated KCa1.1 is required for RABV infection.

### KCa1.1 regulates the internalization of RABV

Since KCa1.1 is primarily expressed at the plasma membrane [32], we hypothesized that it may be involved in RABV entry, which occurs at the cell surface. The process of virus entry involves attachment to the cell surface and internalization into the cells. We therefore first investigated whether KCa1.1 affects RABV binding in N2a, HEK293, and SK cells. KCa1.1-silenced N2a and HEK293 cells, as well as paxilline-treated or control N2a, HEK293, and SK cells, were incubated with ERA-EGFP (MOI 5) at 4°C for 1 h., and then unbound viruses were removed immediately by washing with cold PBS (pH 7.2). The viral RNA (vRNA) levels of the attached viruses were quantified by qPCR. We found that the viruses bound to the KCa1.1-silenced and paxilline-treated cells were comparable to those bound to the control cells in all three cell lines (Fig 2A and 2C), indicating that KCa1.1 has no effect on RABV binding.

**Fig 2:**
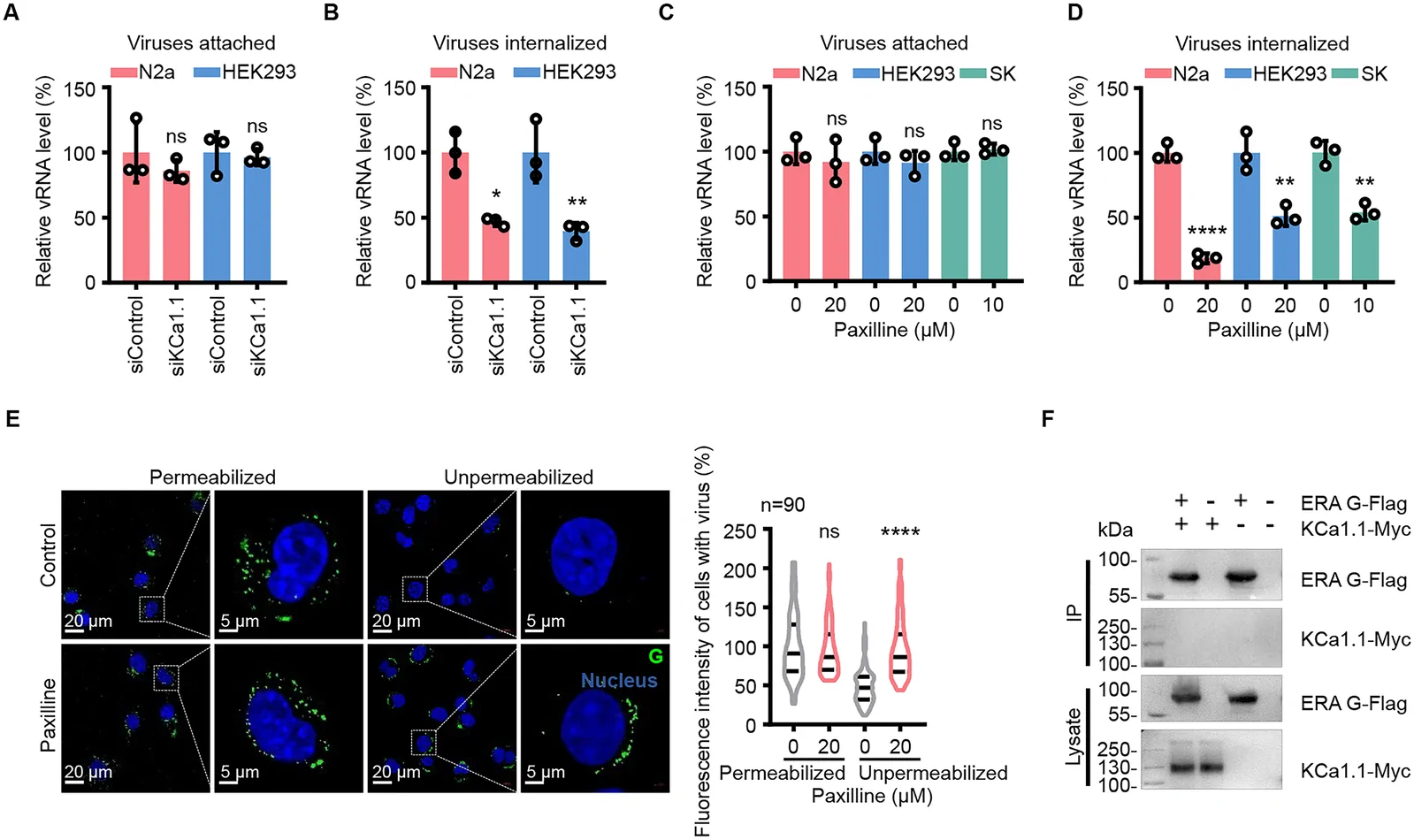
KCa1.1 regulates the internalization of RABV. **(A)** KCa1.1 knockdown did not affect RABV binding to N2a and HEK293 cells. **(B)** KCa1.1 knockdown significantly decreased RABV internalization in N2a and HEK293 cells. **(C)** Paxilline treatment did not affect RABV binding to N2a, HEK293, and SK cells. **(D)** Paxilline treatment significantly decreased RABV internalization in N2a, HEK293, and SK cells. **(E)** A microscopy-based assay showed that paxilline treatment increased the number of RABV particles on the cell surface (unpermeabilized conditions) but not the total number of particles (permeabilized conditions). Nucleus (blue), viral G protein (green). Scale bar, 20 μm and 5 μm. The solid line represents the median and dashed lines represent quartiles of the data in the violin graphs. **(F)** Co-immunoprecipitation assay showed no direct interaction between KCa1.1 and RABV **G.** Data shown in A–E are means  ±  s.d. (n  =  3 biologically independent experiments). Statistical analysis was performed using unpaired, two-tailed Student’s t-test. ns, not significant. * P <  0.05; ** P  <  0.01; **** P  <  0.0001.

We next investigated whether KCa1.1 had an effect on the internalization of RABV in cells. Post-binding, KCa1.1-silenced N2a and HEK293 cells, as well as paxilline-treated or control N2a, HEK293, and SK cells, were shifted to 37℃ for 2 h to allow the entry of bound viruses, respectively. The viruses on the cell surface were removed by washing with trypsin before lysing for qPCR of RABV. The results showed that the vRNA levels in KCa1.1-silenced or paxilline-treated cells were significantly reduced compared to the control cells across all three cell lines (Fig 2B and 2D). The result indicates that activated KCa1.1 is involved in the internalization of RABV. To confirm the role of KCa1.1 in the internalization of RABV, we performed a microscopy-based assay. This assay quantitatively detected RABV G antibody-labeled viruses under permeabilized or unpermeabilized conditions following an internalization period in paxilline-treated or control N2a cells. The fluorescence intensity of each cell was measured, where the intensity under unpermeabilized conditions indicated the number of viral particles that did not enter cells, and the intensity under permeabilized conditions indicated the total number of viral particles. We found that the fluorescence intensity in paxilline-treated N2a cells was higher than that of control cells under unpermeabilized conditions, while was comparable under permeabilized conditions (Fig 2E). These results demonstrate that activated KCa1.1 is involved in the internalization of RABV.

RABV G is responsible for the entry process of RABV [8]. Therefore, we further tested whether KCa1.1 interacts with RABV G by performing a co-immunoprecipitation assay in ERA G-Flag and KCa1.1-Myc-co-transfected HEK293 cells. We found that KCa1.1 did not interact with RABV G (Fig 2F), indicating that other host factors are involved in KCa1.1-mediated RABV entry.

### Ca_v_1.2 interacts with KCa1.1, and is important for RABV infection

A previous study demonstrated that KCa1.1 is assembled into macromolecular complex with the voltage-gated calcium channels Ca_v_1.2, Ca_v_2.1, and Ca_v_2.2, encoding by CACNA1C, CACNA1A, and CACNA1B, respectively [33]. The Ca^2+^ influx through these activated calcium channels can activate KCa1.1 [33]. We found that only Ca_v_1.2 was screened out in our previous screen. And the result of the co-immunoprecipitation assay showed that Ca_v_1.2 interacts with KCa1.1 (Fig 3A). Therefore, we chose Ca_v_1.2 for study. We performed infection assays in Ca_v_1.2-silenced N2a cells or HEK293 cells to test whether Ca_v_1.2 is required for RABV infection. qPCR analysis confirmed that Ca_v_1.2 mRNA expression was significantly decreased in both siCa_v_1.2-transfected cells at 48 h post-transfection (Fig 3B). We found that knockdown of Ca_v_1.2 expression significantly decreased the viral titers at both 24 h and 48 h p.i. (Fig 3C and 3D), suggesting that Ca_v_1.2 is required for RABV infection. We confirmed this result with diltiazem, a benzothiazepine-class L-type calcium channel blocker that inhibits Ca_v_1.2 activity by binding to an intracellularly accessible site within the transmembrane region of the Ca_v_1.2α1C subunit and stabilizing the inactivated state of the channel [34–36]. N2a, HEK293, and SK cells were pre-treated with various concentrations of diltiazem, and then infected with ERA-EGFP (MOI 0.01). The viral titers of the supernatants were measured at 48 h p.i.. We found that diltiazem treatment did not affect cell viability in any of the three cell lines at the highest concentration used and blocked RABV infection in dose-dependent manner (Fig 3E-H). Additionally, we performed a time-course assay using a fixed concentration of diltiazem. N2a or HEK293 cells were pre-treated with diltiazem at a concentration of 200 μM, and viral titers in the supernatants were determined at 24 h, 48 h, and 72 h p.i., whereas SK cells were pre-treated with 40 μM diltiazem and viral titers were determined at 24 h and 48 h p.i. The results showed that diltiazem treatment inhibited RABV infection at the corresponding tested time points (Fig 3I-3K). Ca_v_1.2 antagonists can be categorized into three distinct structural classes: dihydropyridines, benzothiazepines, and phenylalkylamines, which exert their effects by binding to different sites on the Ca_v_1.2 channel and blocking calcium currents [37]. We further selected four FDA-approved Ca_v_1.2 antagonists to test their antiviral efficacy: verapamil, a phenylalkylamine; and nifedipine, nicardipine, and nimodipine, which are dihydropyridines. The results demonstrated that treatment with all four drugs had no effect on HEK293 cell viability but significantly inhibited RABV infection (S1A and S1B Fig). These results collectively demonstrate that activated Cav1.2 is important for RABV infection.

**Fig 3:**
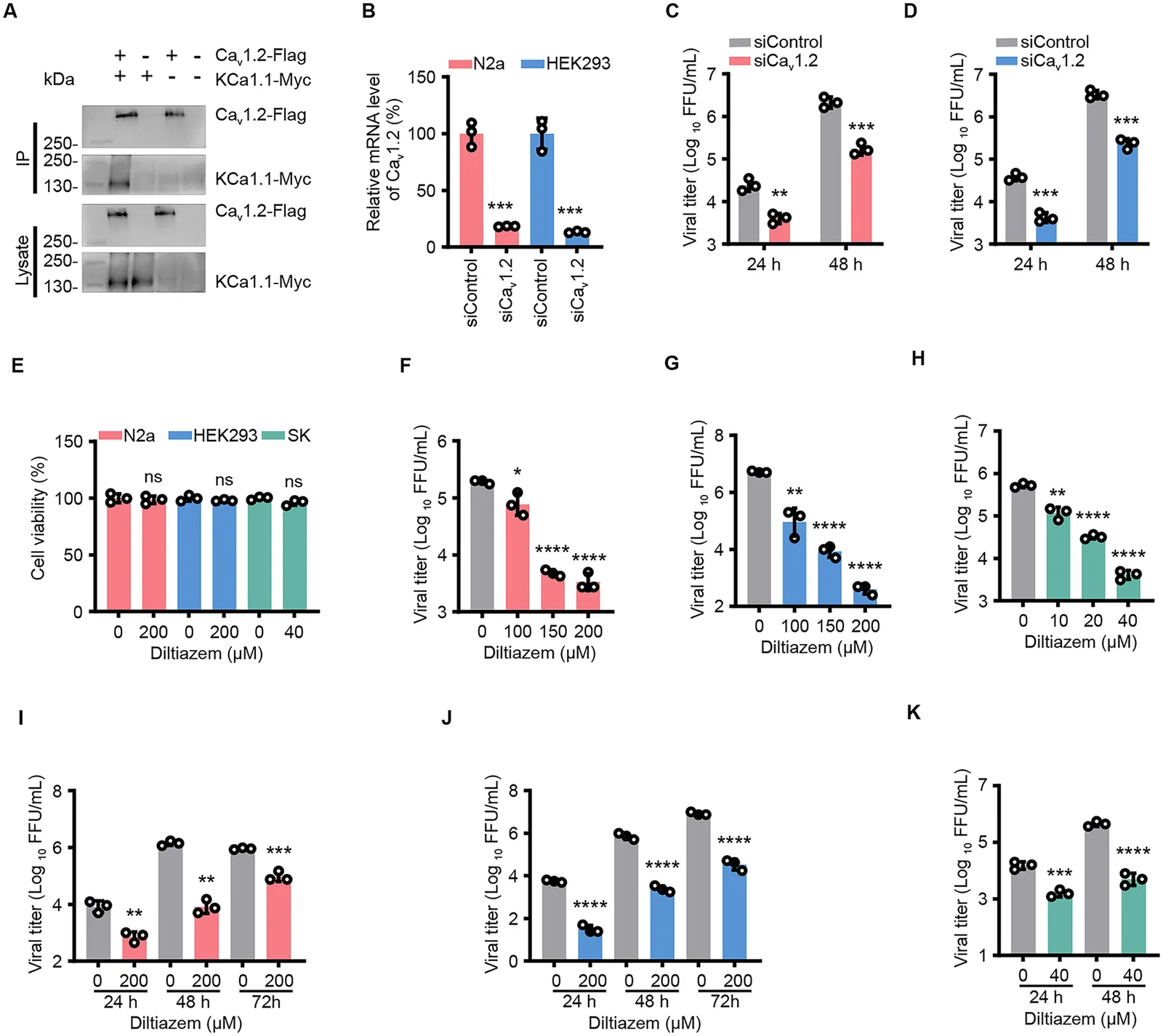
Ca_v_1.2 interacts with KCa1.1, and is important for RABV infection. **(A)** Co-immunoprecipitation assay showed a specific interaction between Ca_v_1.2 and KCa1.1. **(B)** Ca_v_1.2 knockdown significantly reduced Ca_v_1.2 mRNA levels in N2a cells and HEK293 cells. **(C and D)** Ca_v_1.2 knockdown significantly reduced RABV replication in N2a and HEK293 cells. **(E)** Diltiazem treatment did not affect the viability of N2a, HEK293, and SK cells. **(F–H)** Diltiazem treatment significantly inhibited RABV replication in a dose-dependent manner in N2a, HEK293, and SK cells. **(I and J)** Diltiazem treatment reduced RABV replication at 24 h, 48 h, and 72 h post-infection in N2a and HEK293 cells. **(K)** Diltiazem treatment reduced RABV replication at 24 h and 48 h post-infection in SK cells. The data shown in B–K are means  ±  s.d. (n  =  3 biologically independent experiments). Statistical analysis was performed using unpaired, two-tailed Student’s t-test. ns, not significant. ** P  <  0.01; *** P  <  0.001; **** P  <  0.0001.

### Ca_v_1.2 facilitates the internalization of RABV

We next explored whether Ca_v_1.2 is involved in the internalization of RABV. We performed viral binding and internalization assays in both N2a cells and HEK293 cells as described above, and then determined the vRNA levels by using qPCR. We found that the amount of virus bound to Ca_v_1.2-silenced cells was comparable to that bound to control cells (Fig 4A), indicating that Ca_v_1.2 does not affect RABV binding to the cell surface. However, the vRNA levels inside Ca_v_1.2-silenced cells were significantly lower than those in control cells (Fig 4B), suggesting that Ca_v_1.2 plays a crucial role in the internalization of RABV. To test whether the activation of Ca_v_1.2 is required for the internalization of RABV, we explored the effect of diltiazem treatment on RABV entry by using qPCR in N2a, HEK293, and SK cells. We found that diltiazem treatment significantly decreased the internalization of RABV, while having no effect on virus binding (Fig 4C and 4D), indicating that the activation of Ca_v_1.2 is required for the internalization of RABV.

**Fig 4:**
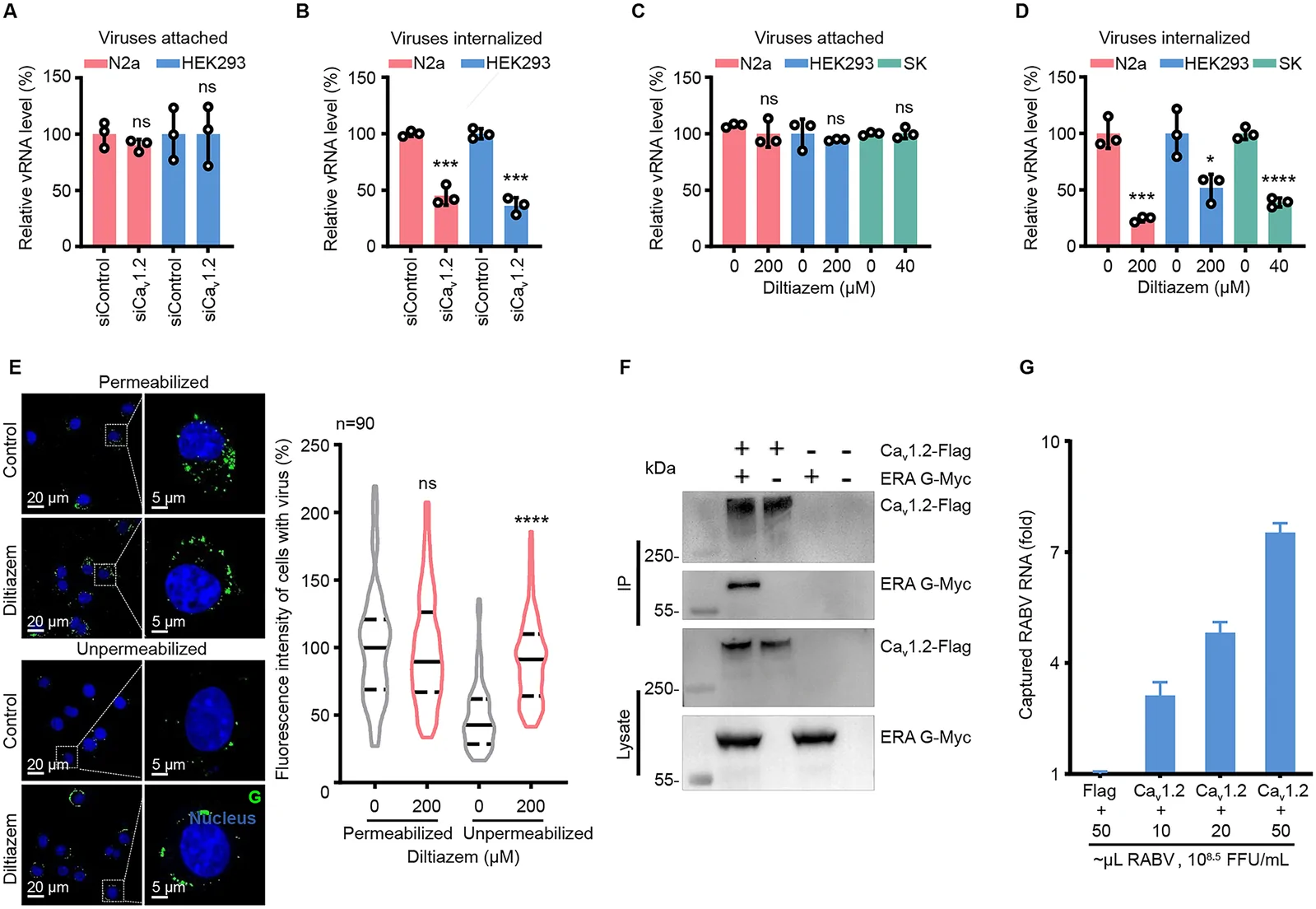
Ca_v_1.2 facilitates the internalization of RABV. **(A)** Ca_v_1.2 knockdown did not affect RABV binding to N2a and HEK293 cells. **(B)** Ca_v_1.2 knockdown significantly decreased RABV internalization in N2a and HEK293 cells. **(C and D)** Diltiazem treatment reduced RABV internalization but not binding in N2a, HEK293, and SK cells. **(E)** Microscopy-based assay revealed that diltiazem-treated N2a cells had more RABV particles on the cell surface under unpermeabilized conditions, but the total number of particles was comparable under permeabilized conditions. Nucleus (blue), viral G protein (green). Scale bar, 20 μm and 5 μm. The solid line represents the median and dashed lines represent quartiles of the data in the violin graphs. **(F)** Co-immunoprecipitation assay showed a specific interaction between Ca_v_1.2 and RABV **G. (G)** Pull-down assay showed a specific interaction between Ca_v_1.2 and RABV particles. The data shown in A–E are means  ±  s.d. (n  =  3 biologically independent experiments). Statistical analysis was performed using the unpaired, two-tailed Student’s t-test. ns, not significant. * P <  0.05; *** P  <  0.001; **** P <  0.0001.

We confirmed the importance of Ca_v_1.2 on the internalization of RABV by performing a microscopy-based assay as described above in diltiazem- or mock-treated N2a cells. The results showed that the fluorescence intensity in diltiazem-treated N2a cells was higher than that of control cells under unpermeabilized conditions, while was comparable under permeabilized conditions (Fig 4E), confirming that Ca_v_1.2 is required for the internalization of RABV.

We further investigated whether Ca_v_1.2 interacts with RABV G by performing a co-immunoprecipitation assay. Ca_v_1.2-Flag was co-expressed with ERA G-Myc in plasmid-transfected HEK293 cells. Immunoblotting for ERA G-Myc demonstrated that Ca_v_1.2 interacts with ERA G specifically (Fig 4F). We further examined the interaction between Ca_v_1.2 and RABV using Ca_v_1.2 lysates and purified RABV particles. RABV particles were efficiently captured by Ca_v_1.2 using protein G agarose, with the amount of captured virus increasing in a dose-dependent manner (Fig 4G). These above results demonstrated that the activation of Ca_v_1.2 is involved in the internalization of RABV.

### Activated KCa1.1 and Ca_v_1.2 regulate F-actin polymerization during RABV entry

A previous study demonstrated that KCa1.1 can promote influenza virus entry through regulating the F-actin polymerization [26]. We next investigated whether Ca_v_1.2 and KCa1.1 exploit a similar mechanism to regulate RABV entry. To test this concept, we evaluated F-actin polymerization in HEK293 control cells, HEK293 cells treated with diltiazem or paxilline, and Ca_v_1.2- or KCa1.1-knockdown HEK293 cells. After being incubated with ERA-EGFP (MOI 5) at 4°C for 1 h and then at 37°C for 30 min, the cells were fixed and stained with the F-actin dye phalloidin-488 for 1 h. Fluorescence intensity was then measured. Control HEK293 cells that were not incubated with ERA-EGFP were used as a negative control. The fluorescence intensity of phalloidin in control HEK293 cells incubated with ERA-EGFP was higher than in those not incubated with ERA-EGFP, suggesting that RABV entry can induce F-actin polymerization. We found that the fluorescence intensity of phalloidin in the Ca_v_1.2- or KCa1.1-knockdown cells and in cells treated with diltiazem or paxilline was lower than that in the HEK293 control cells incubated with ERA-EGFP (Fig 5A and 5B). The results demonstrate that both Ca_v_1.2 and KCa1.1, when activated, positively regulate F-actin polymerization.

**Fig 5:**
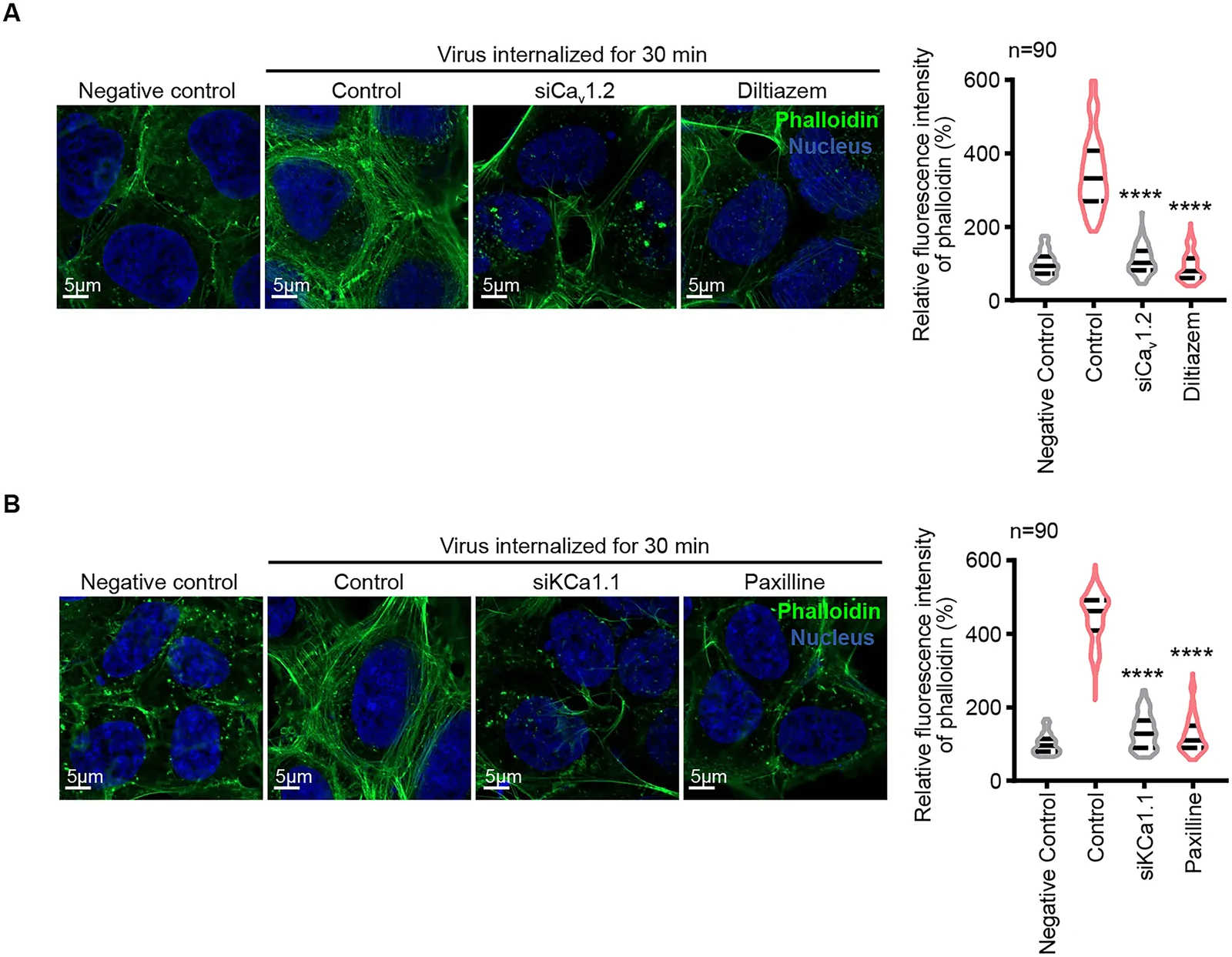
Activated KCa1.1 and Ca_v_1.2 regulate F-actin polymerization during RABV entry. **(A and B)** Knockdown of Ca_v_1.2 or KCa1.1 and treatment with diltiazem or paxilline significantly reduced F-actin polymerization in RABV-internalized HEK293 cells. The fluorescence intensity of phalloidin was quantified using ZEN software. Nucleus (blue), phalloidin (green). Scale bar: 5 μm. Statistical analysis was performed using the unpaired, two-tailed Student’s t-test. **** P <  0.0001.

### Targeting Ca_v_1.2 inhibits RABV infection in mice

There is no drug proven to prevent RABV-induced death. Our *in vitro* studies have demonstrated that diltiazem, an FDA-approved drug [28], can inhibit the internalization of RABV. Does diltiazem have a potential therapeutic effect on RABV infection *in vivo*? To address this question, we first tested the toxicity of various concentrations of diltiazem in mice. We found that diltiazem treatment had no obvious effect on the weight of mice when intramuscularly inoculated with diltiazem at a dose of 25 mg/kg or 50 mg/kg once daily for seven days (Fig 6A). We next performed RABV infection with the RABV street virus GX/09 strain in mice. 6-week-old C57BL/6J mice were inoculated intramuscularly with RABV GX/09 at a dose of 10 50% mouse lethal dose (MLD_50_). Six hours later, the mice were intramuscularly inoculated with PBS or diltiazem at a dose of 25 mg/kg or 50 mg/kg, and then once daily for the following six days. Six mice from each of the PBS-treated and 50 mg/kg diltiazem-treated groups were euthanized on days 4 and 6 post-inoculation, respectively, and their brains were collected for quantification of viral RNA levels by qPCR. Three mice from the PBS-treated and 50 mg/kg diltiazem-treated groups were euthanized on day 8 post-inoculation, and their brains were collected for detection of RABV antigen by immunohistochemistry. The remaining 19 mice in each group were monitored for neurological symptoms and survival for 28 days (Fig 6B).

**Fig 6:**
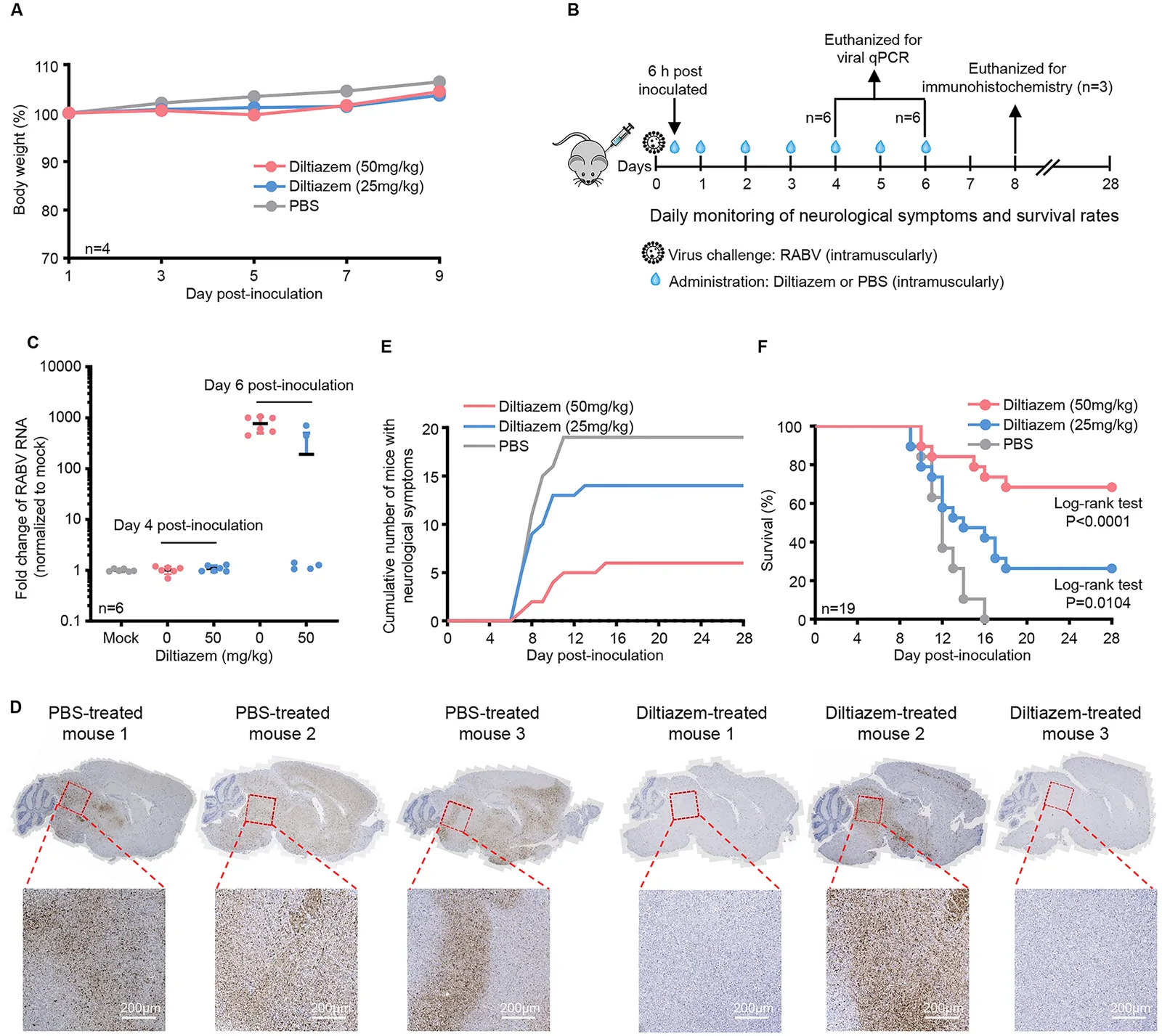
The FDA-approved drug diltiazem inhibits RABV infection in mice. **(A)** Diltiazem treatment did not affect the weight of mice. **(B)** Experimental protocol for diltiazem treatment in C57BL/6J mice. **(C and D)** C57BL/6J mice were intramuscularly administered Diltiazem (50 mg/kg or 25 mg/kg) or PBS starting 6 h after infection with RABV-GX/09, followed by daily administration for the next six days. **(C)** Brain viral RNA levels in diltiazem- or PBS-treated mice infected with RABV GX/09 were quantified by qPCR at 4 and 6 days post-inoculation. **(D)** Immunohistochemical analysis of RABV antigen in brain tissues collected from diltiazem- or PBS-treated mice at day 8 post-inoculation. Neurological symptoms (E) and survival (F) of the mice (n = 19) were monitored for 28 days post-inoculation. Statistical analysis of survival rates between PBS-treated and Diltiazem-treated mice was performed using the Log-rank (Mantel-Cox) test.

qPCR analysis showed that no viral RNA was detected in the brains of mice at day 4 post-inoculation. At day 6 post-inoculation, viral RNA was detected in all PBS-treated mice (6/6), whereas only 2 out of 6 diltiazem-treated mice exhibited detectable viral RNA. The viral RNA levels detected in these two diltiazem-treated mice were comparable to those in PBS-treated mice (Fig 6C). Immunohistochemical analysis of brain tissues showed RABV antigen staining in all three PBS-treated mice, while detectable antigen signals were observed in only one out of three diltiazem-treated mice (Fig 6D).

All 19 PBS-treated mice died within 9–16 days of infection, resulting in 100% mortality. In the group treated with the 25 mg/kg diltiazem, 14 mice died within 9–18 days of infection and 5 mice survived, yielding about 26% survival rate. In the group treated with 50 mg/kg diltiazem, 6 mice died within 10–18 days of infection and 13 mice survived, leading to about 70% survival rate. The surviving mice in the diltiazem treatment group remained completely asymptomatic throughout the 28-day observation period. All of the dead mice displayed typical rabies disease signs, such as body tremors and hind limb paralysis (Fig 6E and 6F). The results demonstrate that diltiazem treatment significantly inhibits RABV infection *in vivo* and improves the survival rate of RABV-infected mice in a dose-dependent manner, suggesting its potential as a therapeutic drug against RABV infection.

Given that diltiazem has been reported to cross the blood–brain barrier (BBB) [38, 39], we next investigated whether diltiazem inhibits RABV entry into the CNS or viral replication within the CNS. To address this question, mice were administered diltiazem (50 mg/kg) or PBS intramuscularly on days 6 and 7 post-inoculation, when RABV replication was already underway in the brain. Brain tissues were collected at day 8 post-inoculation and viral RNA levels were quantified using qPCR. No significant reduction in brain viral RNA levels was observed in the diltiazem-treated group relative to the PBS control group (S2 Fig). These data indicate that diltiazem-mediated protection is unlikely to arise from direct suppression of RABV replication in the CNS after viral neuroinvasion.

## Discussion

In this study, we found that the calcium channel Ca_v_1.2 and the K^+^ channel KCa1.1 are important for the internalization process of the RABV. Activated Ca_v_1.2 and KCa1.1 promote the internalization of RABV through regulating F-actin polymerization. Additionally, we discovered a specific interaction between Ca_v_1.2 and the RABV G, which was not observed for KCa1.1. A previous study demonstrated that activated Ca_v_1.2 interacts with and activates KCa1.1 [33]. We, therefore, propose a sequential model for the internalization of RABV mediated by these channels. RABV interacts with Ca_v_1.2, triggering its activation. Subsequently, the activated Ca_v_1.2 interacts with and activated KCa1.1. The activated KCa1.1 then facilitates the internalization of RABV through regulating F-actin polymerization.

There are three categories of exposure to suspected rabid animals, with category III exposures requiring the most intensive post-exposure prophylaxis [40]. This includes thorough wound cleaning, deep irrigation with a potent antiseptic agent, administration of the first dose of rabies vaccine, and the use of rabies immunoglobulin or monoclonal antibodies to neutralize RABV before it enters CNS [41]. Unfortunately, despite the high efficacy of rabies post-exposure prophylaxis, human deaths still occur due to the lack of or delay in receiving timely treatment, particularly after severe exposures [42]. Most rabies deaths occur in poor rural populations in Africa and Asia where the out-of-pocket cost of rabies post-exposure prophylaxis can be catastrophic [43]. Furthermore, a global shortage of rabies immunoglobulin and monoclonal antibodies exacerbates the issue [6]. It has been estimated that only 1–10% of category III patients receive the recommended rabies post-exposure prophylaxis [44]. This highlights a pressing public health need for safe, effective, and affordable therapeutic alternatives. In this study, we found that when administered intramuscularly at a dose of 50 mg/kg/day, 6 h post-RABV exposure, diltiazem conferred a survival rate of approximately 70% in RABV-infected mice. The oral 50% lethal dose (LD_50_) is 415–740 mg/kg in mice [45], and the intravenous LD_50_ is 60 mg/kg in mice [45], both higher than the doses used in this study. Diltiazem was approved in the US in 1982 and widely used in clinical practice for many indications, including atrial arrhythmia, hypertension, and angina [34, 46, 47]. Clinically, diltiazem is administered orally or intravenously. The oral dose for adults is about 120–480 mg/day and the dose for continuous intravenous infusion is about 120–240 mg/day [48]. Based on body surface area normalization following FDA guidelines, the 50 mg/kg dose in mice corresponds to a human equivalent dose (HED) approximately 4 mg/kg. For an adult weighing 60 kg, this HED corresponds to approximately 240 mg/day, which falls within the commonly used therapeutic range of diltiazem in humans (~240 mg/day). Given that diltiazem is an affordable drug and no supply problem, therefore, diltiazem can be used as a promising alternative for rabies post-exposure prophylaxis in resource-limited areas, offering both a temporary solution when immunoglobulin and antibodies are lacking and a potential enhancement when used alongside them.

Understanding how diltiazem confers protection against RABV infection is important for elucidating its antiviral mechanism and evaluating its therapeutic potential. Several possible mechanisms may account for the protective effect of diltiazem *in vivo*. One possibility is that diltiazem primarily inhibits RABV entry at peripheral infection sites, thereby limiting initial viral dissemination and subsequent retrograde axonal transport to the CNS. Consistent with this possibility, early administration of diltiazem significantly reduced viral RNA accumulation in the brain and decreased RABV antigen distribution (Fig 6C and 6D), suggesting that blocking viral entry before neuroinvasion represents a major contributor to its protective effect. Another possibility is that diltiazem may directly inhibit RABV replication within the CNS after crossing the BBB, as diltiazem is a lipophilic calcium channel blocker that has been reported to penetrate the BBB [38, 39]. However, under the current treatment schedule and experimental conditions, delayed administration of diltiazem after RABV replication had been initiated in the brain did not significantly reduce brain viral RNA levels (S2 Fig), suggesting that the antiviral effect of peripherally administered diltiazem is unlikely to be primarily mediated by direct inhibition of established RABV infection in the CNS. Nevertheless, the efficiency of diltiazem penetration across the BBB and whether its concentration within the CNS reaches a level sufficient to inhibit RABV replication remain unclear and require further investigation.

Ca_v_1.2 and KCa1.1 have been implicated in the entry process of various viruses [18]. Our previous studies demonstrated that activated Ca_v_1.2 is required for the binding and internalization of SARS-CoV-2 [22], and activated KCa1.1 is involved in the endocytosis of influenza virus [26]. A study by Fujioka et al. reported that activated Ca_v_1.2 facilitates influenza virus entry into mammalian cells [23]. The present study further elucidates the roles of Ca_v_1.2 and KCa1.1 in the entry process of RABV. It seems like Ca_v_1.2 and KCa1.1 have a conserved role in the internalization of various viruses. Diltiazem treatment also inhibits the infection of the human rotavirus virus, porcine reproductive and respiratory syndrome virus infection, and porcine deltacoronavirus [49–51]. Whether KCa1.1 is involved in the infection of these Ca_v_1.2-dependent viruses requires further study. Ca_v_1.2 and KCa1.1 could be targets for the development of broad-spectrum antiviral agents.

Ion channels represent a promising class of drug targets. Our results demonstrate that KCa1.1 and Ca_v_1.2 are two ion channels that are important for RABV infection. Knockdown of Ca_v_1.2 or KCa1.1 led to reducing the internalization of RABV in cells. However, for KCa1.1, there are currently no approved drugs that target it. Despite this, we have shown that the KCa1.1 antagonist paxilline significantly inhibits RABV infection in cells, suggesting the potential of KCa1.1 as a promising target for the development of antiviral drugs against RABV. For Ca_v_1.2, both *in vitro* and *in vivo* assays have demonstrated that the Ca_v_1.2 antagonist diltiazem is a promising candidate for anti-rabies therapy. Ca_v_1.2 antagonists are classified into three groups: dihydropyridines, benzothiazepines, and phenylalkylamines, which target distinct sites on the Ca_v_1.2 channel. Besides diltiazem, other Ca_v_1.2 antagonists, including verapamil, a phenylalkylamine, and the dihydropyridines nifedipine, nicardipine, and nimodipine, also inhibited RABV infection *in vitro*. Further studies are required to evaluate their protective effects against RABV infection *in vivo*.

## Materials and methods

### Ethics statement

Animal studies were carried out in strict accordance with the recommendations in the Guide for the Care and Use of Laboratory Animals of the Ministry of Science and Technology of the People’s Republic of China. The protocols were approved by the institutional Committee on the Ethics of Animal Experiments (approval number IACUC-230221–02-GJ, IACUC-260625–01-GJ).

### Mice, cells, and viruses

Wild-type C57BL/6J mice were purchased from Vital River Laboratories (Beijing, China). Mice were housed in ventilated cages (maximum of eight mice per cage) under controlled humidity, temperature, and light.

HEK293 cells (ATCC, CRL-1573) and N2a cells (ATCC, CCL-131) were maintained in DMEM supplemented with 10% fetal bovine serum (FBS). Human neuroblastoma cells SK-N-SH (SK cells) (ATCC HTB-11) were maintained in MEM/EBSS supplemented with 10% FBS. BHK-21 cells (ATCC, CCL-10) were maintained in DMEM containing 5% FBS. The cells were cultured at 37°C with 5% CO2.

The RABV ERA strain, originally devrived by Rokitnicki Abelseth [52], was obtained from the China Veterinary Culture Collection (CVCC) [53]. Recombinant ERA expressing EGFP (ERA-EGFP) was generated as previously described [54]. Rabies street virus GX/09 was passaged in mouse brain, and the intramuscular 50% lethal dose (LD_50_) in mice was determined prior to the challenge study.

### Antibodies, reagents, and chemicals

Mouse anti-Flag-tag pAb (CN: A00187, 1:1000) and rabbit anti-Myc-tag pAb (CN: A00172, 1:1000) were from Genscript. Goat anti-mouse IgG coupled with Alexa Fluor 488 (CN: ab150113, 1:1000) and Phalloidin iFluor 488 (CN: ab176753, 1:2000) were from Abcam. Goat anti-rabbit IgG coupled with HRP (CN: 31460, 1:5000) and goat anti-mouse IgG coupled with HRP (CN: 31430, 1:5000) were from Invitrogen. Opti-MEM (CN: 31985070), Lipofectamine RNAiMAX Transfection Reagent (CN: 13778150), 4’,6-diamidino-2-phenylindole (DAPI) (CN: D3571), Halt Protease Inhibitor Cocktail (CN: 87786), and Pierce IP Lysis Buffer (CN: 87788) were from Invitrogen. NP-40 Lysis Buffer (CN: P0013F) was from Beyotime. Anti-Flag-tag antibody-conjugated agarose beads (CN: A2220) were from Sigma-Aldrich. CellTiter-Glo kit (CN: G9242) was from Promega. Diltiazem (CN: D2521-1G) was from Sigma-Aldrich. ExFect Transfection Reagent (CN: T101-02), Phanta Super-Fidelity DNA Polymerase (CN: P501-d2) and ChamQ Universal SYBR qPCR Master Mix (CN: Q711-03) were from Vazyme. First Strand cDNA Synthesis Kit (CN: FSK-101) was from TOYOBO. PVDF membrane (CN: ISEQ00010) and Immobilon Crescendo Western HRP substrate (CN: WBLUR0500) were from Merck-Millipore. TRIzol (CN: 15596018) was from Thermo Fisher Scientific. Benzonase (CN: C2001) was from HaiGene. The 4%–12% ExpressPlus PAGE Gel (CN: M41210C) was from Genscript. Protein G agarose (CN: 11243233001) was from Roche. Paxilline (CN: B6920) was from Apexbio.

### Plasmids

The KCa1.1 and Ca_v_1.2 over-expressing plasmids were constructed by inserting the cDNA of human KCa1.1 and Ca_v_1.2 into the mammalian expression vector pCAGGS, with Myc or Flag tags at the C-terminus. The G gene of the ERA strain was cloned into the pCAGGS vector with Flag or Myc tags at the C-terminus to generate ERA G-Flag and ERA G-Myc, respectively. All constructs were confirmed by sequencing.

### Cell viability assay

Cell viability was determined using the CellTiter-Glo kit according to the manufacturer’s instructions. HEK293, N2a, or SK cells treated with chemicals at the indicated concentrations were cultured in opaque 96-well plates for the specified times. Then, 100 μL of CellTiter-Glo reagent was added per well, and the cells were incubated for 10 min at room temperature. Luminescence was measured using a GloMax 96 Microplate Luminometer.

### Virus infection assay

Cells were infected with the indicated virus in 100 μL of inoculum for 1 h at 37°C. After infection, cells were washed three times with PBS, followed by the addition of 500 μL of DMEM or MEM/EBSS supplemented with 2% FBS for further incubation and analysis.

### Quantitative PCR (qPCR)

To quantify mRNA levels of Ca_v_1.2, KCa1.1, or viral RNA (vRNA), total RNA was extracted using TRIzol reagent. cDNA was synthesized with the First Strand cDNA Synthesis Kit. The qPCR reactions were carried out with ChamQ Universal SYBR qPCR Master Mix using specific primer. The qPCR was performed on an Applied Biosystems QuantStudio 5 Real-Time PCR System (Thermo Fisher). The 2^-∆∆Ct^ method was used to calculate relative gene expression levels, with 28S rRNA as the internal control. The qPCR primers used were as follows: KCa1.1 (human), forward (5’-GGAATGGGAGACGCTTCATAA-3’) and reverse (5’-TCTGATTGGCTGACAGGATAAC-3’); KCa1.1 (mouse), forward (5’-GCATTGGTGCCCTCGTAATA-3’) and reverse (5’-GCCAAAGTAGAGGAGGAAGAAC-3’); Ca_v_1.2 (human), forward (5’-GTCACCTTTCAGGAGCAGGG-3’) and reverse (5’-AGGTGGAGTTGACCACGTACC-3’); Ca_v_1.2 (mouse), forward

(5’-CGTGCAGCAGTTAAGTCCAA-3’) and reverse (5’-CTTTATTGGCTGTGTCTTGC-3’); and RABV N, forward (5’-ATGAAGACTGTTCAGGACTGGTAT-3’) and reverse (5’-CCCTGGCTCAAACATTCTTCTTA-3’); GX/09 N, forward (5’-AACATTGCGGACAGGATAGAG-3’) and reverse (5’-GTTCGGTATGGTACTCCAGTTAG-3’); β-actin (mouse), forward (5’-TGTTACCAACTGGGACGACA-3’) and reverse (5’-GGGGTGTTGAAGGTCTCAAA-3’).

### RNA interference (RNAi) and overexpression assay

RNAi assays were conducted in 24-well plates. Briefly, 120 μL of Opti-MEM containing 1 μL of Lipofectamine RNAiMAX Transfection Reagent was added to wells pre-arrayed with siRNAs. After a 30-minute incubation at room temperature, HEK293 cells (8 × 10^3^ cells/well) and N2a cells (7.5 × 10^3^ cells/well) were added in a volume of 500 μL. The siRNA-transfected HEK293 and N2a cells were maintained at 37°C for 60 and 72 h, respectively, before infection with ERA-EGFP.

Overexpression assays were performed in 6-well plates. HEK293 cells (1 × 10⁶ cells/well) were incubated similarly, with 200 μL of Opti-MEM containing 2 μL of ExFect Transfection Reagent. After a 15-minute incubation at room temperature, the transfection mixture was added to the cells. Plasmid-transfected HEK293 cells were maintained at 37°C for 48 h prior to Co-immunoprecipitation assays. The siRNA sequences were as follows: siKCa1.1 (human), Sense: (5’-GUCUUAGAAUGAGUAGCAAtt-3’), Antisense: (5’-UUGCUACUCAUUCUAAGACtg-3’); si KCa1.1 (mouse), Sense: (5’-GAAAUACUACUUGGAAGGAdTdT), Antisense: (5’-UCCUUCCAAGUAGUAUUUCdTdT); siCa_v_1.2 (human), Sense: (5’-GAAACUAUAUCCUACUGAAtt-3’), Antisense: (5’-UUCAGUAGGAUAUAGUUUCca-3’); siCa_v_1.2 (mouse), Sense: (5’-CCAGGGAUGUUAGUCUGUAtt-3’), Antisense: (5’-UACAGACUAACAUCCCUGGaa-3’).

### Drug inhibition assays

For the dose-response assays, HEK293, N2a and SK cells were pre-treated with various concentrations of the drugs for 1 h, followed by infection with ERA-EGFP (MOI 0.01). At 48 h p.i., the supernatants were collected for virus titration in BHK-21 cells.

For the time-course assays, N2a or HEK293 cells were pre-treated with 20 μM paxilline or 200 μM diltiazem, followed by infection with ERA-EGFP (MOI 0.01). At 24 h, 48 h, and 72 h p.i., the supernatants were collected for virus titration in BHK-21 cells. SK cells were pre-treated with 10 μM paxilline or 40 μM diltiazem and infedted with ERA-EGFP (MOI 0.01). At 24 h and 48 h p.i., the supernatants were collected for virus titration in BHK-21 cells.

### Viral binding assay

For the viral binding assay, drug-treated or siRNA-transfected HEK293 cells, N2a cells and SK cells in 24-well plates were infected with ERA-EGFP (MOI 5) at 4°C for 1 h. The cells were then washed three times with cold PBS and lysed to measure the vRNA levels by qPCR.

### Viral internalization assay

For the viral internalization assay, drug-treated or siRNA-transfected HEK293 cells, N2a cells and SK cells in 24-well plates were infected with ERA-EGFP (MOI 5) and maintained at 4°C for 1 h. The cells were then washed three times with cold PBS and incubated at 37°C for 2 h. After incubation, the cells were washed with 0.25% trypsin to remove the virus retained on the surface of the plasma membrane. Finally, the cells were lysed, and the vRNA levels were analyzed by qPCR. For the microscopy-based assay, virus-bound N2a cells were washed three times with cold PBS and maintained at 37°C for 1 h. The cells were fixed, and the cell surface G was analyzed using the confocal laser scanning microscopy assay with the indicated antibodies.

### Western blotting

The plasmid-transfected cells were washed twice with cold PBS and lysed in Pierce IP Lysis Buffer containing Halt Protease Inhibitor Cocktail and Benzonase for 1 h at 4°C. The cell lysates were then centrifuged at 12,000 rpm (or 13,523 g) for 10 minutes at 4°C. The supernatants were collected, mixed with protein sample loading buffer, and boiled for 10 minutes. The protein samples were loaded onto a 4%–12% ExpressPlus PAGE Gel and separated by electrophoresis. The proteins were then transferred onto a PVDF membrane. The PVDF membrane was blocked with 5% skim milk in PBS containing 0.1% Tween-20 (PBST buffer) and incubated with the indicated primary antibody at room temperature for 1 h. After three washes with PBS, the membrane was incubated with the appropriate HRP-conjugated secondary antibody. Following three additional washes with PBST buffer, the target protein bands were visualized using the Immobilon Crescendo Western HRP substrate and detected with the Odyssey infrared imaging system (Li-Cor BioSciences, Lincoln, NE).

### Co-immunoprecipitation

For the Co-immunoprecipitation assay, the cells expressing the relevant proteins were lysed as described in the Western blotting protocol. The supernatants of the cell lysates were first mixed with 60 μL of protein G Agarose and incubated for 4 h at 4°C on a flip shaker. Following centrifugation to remove the protein G beads, the cleared supernatants were incubated with anti-Flag-tag antibody-conjugated agarose beads for 12 h at 4°C on a flip shaker. The beads were then washed five times with cold NP-40 Lysis Buffer to remove any non-specifically bound proteins. After washing, the beads were resuspended in PBS, mixed with protein sample loading buffer, and boiled for 10 minutes. The samples were then subjected to SDS-PAGE and analyzed by Western blotting to detect the co-immunoprecipitated proteins.

### Pull-down assay

This assay was performed as previously described [13]. Briefly, Flag- or Ca_v_1.2-transfected cell lysates were mixed with 50 μL protein G beads at 4°C for 2 h, respectively. After conjugation, the beads were mixed with different amounts of purified RABV particles (10, 20, or 50 μL, FFU/mL = 10^8.5^) in 0.2 mL of PBS. After 2 h, the samples were washed eight times with PBS. Then they were assessed by qPCR analysis to detect the captured vRNA.

### F-actin polymerization assay

For the F-actin polymerization assay, the drug-treated or siRNA-transfected cells were infected with ERA-EGFP (MOI 5) at 37°C for 30 minutes. Following infection, the cells were fixed with 4% paraformaldehyde and washed three times with PBS to remove residual virus and fixative. The cells were then incubated with Phalloidin iFluor 488 for 1 h at room temperature to stain F-actin filaments. After incubation, the cells were washed three times with PBS to remove excess dye. Then, the F-actin polymerization in the cells was analyzed using confocal laser scanning microscopy. The fluorescence intensity of phalloidin was quantified using ZEN software.

### Mouse experiment

Randomization was employed to assign mice to experimental groups. Nineteen 6-week-old C57BL/6J mice were randomly assigned to each group. The mice were intramuscularly inoculated with 10 MLD_50_ of the RABV GX/09 strain in a volume of 0.1 mL PBS. Six hours later, the mice received intramuscular injections of either PBS, 25 mg/kg diltiazem, or 50 mg/kg diltiazem, followed by daily injections for the next six days. Brain tissues were collected for qPCR quantification of viral RNA levels on days 4 and 6 post-inoculation as described above. Brain tissues were collected for immunohistochemical analysis on day 8 post-inoculation as described previously [12]. Neurological symptoms and survival were monitored over a 28-day period.

### Statistics and reproducibility

All quantitative data are presented as the means  ±  standard deviation (s.d.) from at least three independent experiments or replicates. Statistical analyses were performed using GraphPad Prism. An unpaired, two-tailed Student’s t-test was used for comparisons between groups, as indicated in the figure legends. Survival analysis was conducted using the log-rank (Mantel–Cox) method. Differences in means were considered statistically significant at * P  <  0.05; ** P  <  0.01; *** P  <  0.001; and **** P  <  0.0001; ns, not significant. Data distribution was assumed to be normal but was not formally tested. Sample sizes were not predetermined by statistical methods but were based on those used in similar studies. Blinding was applied during data analysis, and no data points or animals were excluded from the analyses.

## Supporting information

S1 Fig FDA-approved Ca_v_1.2 antagonists inhibit RABV infection in cells. (A) HEK293 cells were treated with four FDA-approved Ca_v_1.2 antagonists at indicated concentrations. Cell viability was not significantly affected. (B) All four drugs (verapamil, nifedipine, nicardipine, and nimodipine) significantly reduced RABV replication in HEK293 cells. Data represent means  ±  s.d. (n  =  3 biologically independent experiments). Statistical analysis was performed using unpaired, two-tailed Student’s t-test. ns, not significant. * P  <  0.05; **** P  <  0.0001.
(TIF)

S2 Fig Diltiazem shows no direct anti-RABV activity in the brain post neuroinvasion. Mice were then administered PBS or diltiazem intramuscularly on days 6 and 7 post-inoculation, and brain tissues were collected on day 8 post-inoculation for viral RNA quantification by qPCR. Statistical analysis was performed using the unpaired, two-tailed Student’s t-test. ns, not significant.
(TIF)

S1 Table siRNA information from the 16 genes related to potassium channels screening. (XLSX)

S3 Fig Original blot data. (PDF)

S1 File Original data in the manuscript, Figs 1–6 and S1-S2 Figs. (XLSX)
