# Supplementary material for: Targeting the Cav1.2-KCa1.1 axis prevents rabies virus internalization and protects against lethal infection
Source: PLoS Pathog. 2026 Oct 6;22(10):e1014675. doi: 10.1371/journal.ppat.1014675 (PMC13660984; doi:10.1371/journal.ppat.1014675)

Fig 2F. Co-immunoprecipitation assay showed no direct interaction between KCa1.1 and RABV G.
Captured by Chemiluminescence Imaging system (FM1006).

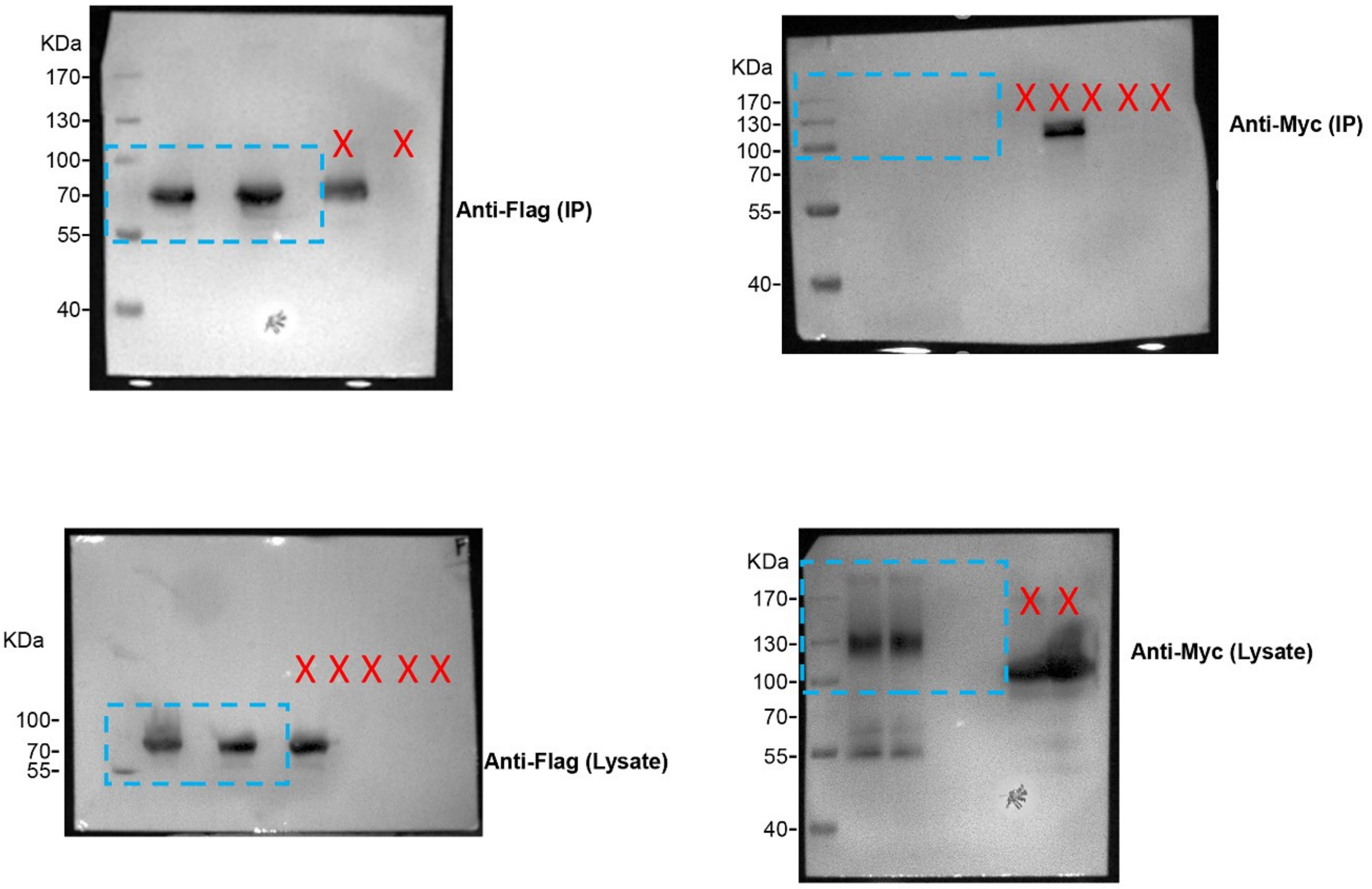

Fig 3A. Co-immunoprecipitation assay showed a specific interaction between $Ca_v1.2$ and KCa1.1.

Captured by Chemiluminescence Imaging system (FM1006).

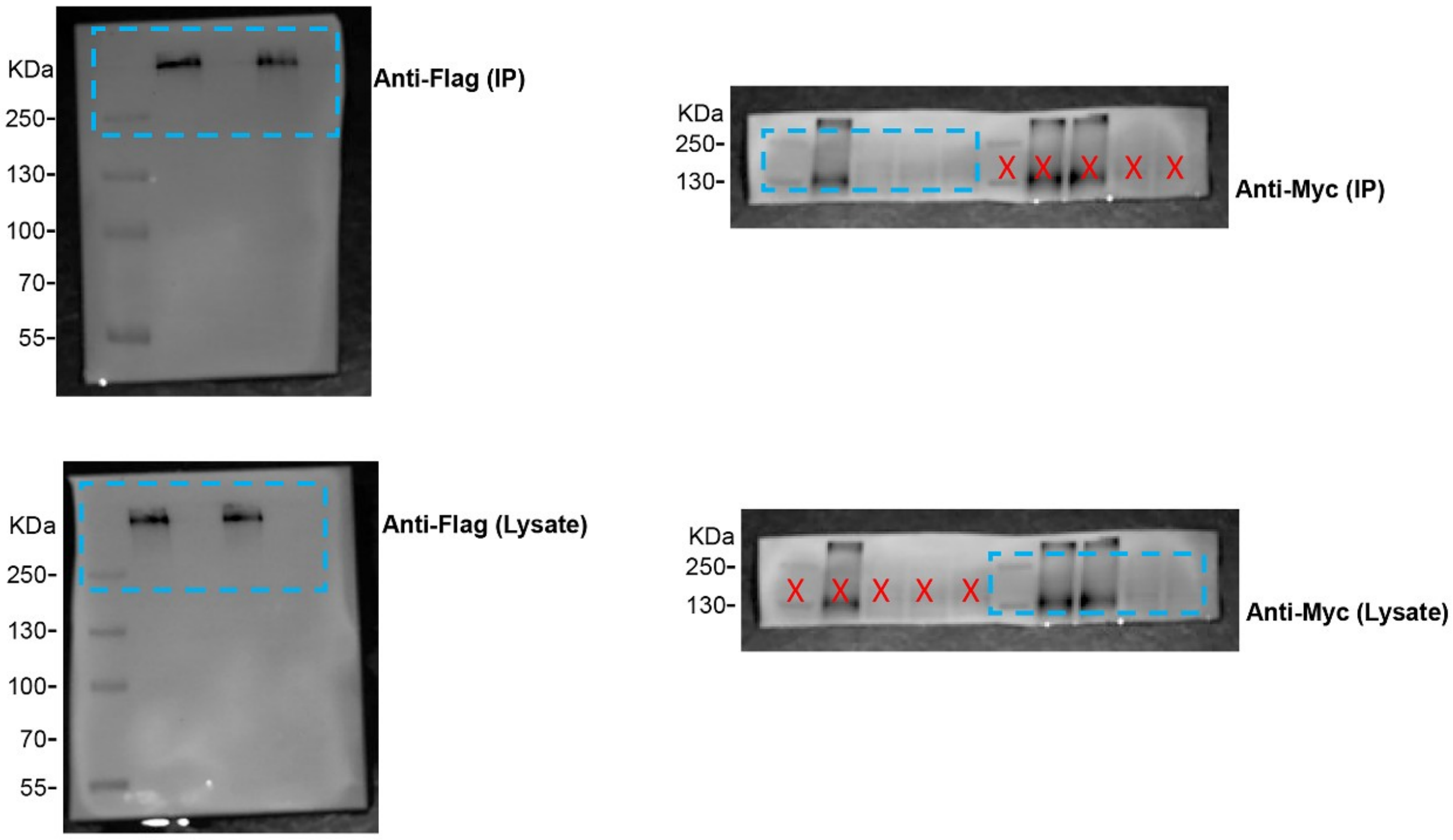

Fig 4F. Co-immunoprecipitation assay showed a specific interaction between Cav1.2 and RABV G.
Captured by Chemiluminescence Imaging system (FM1006).

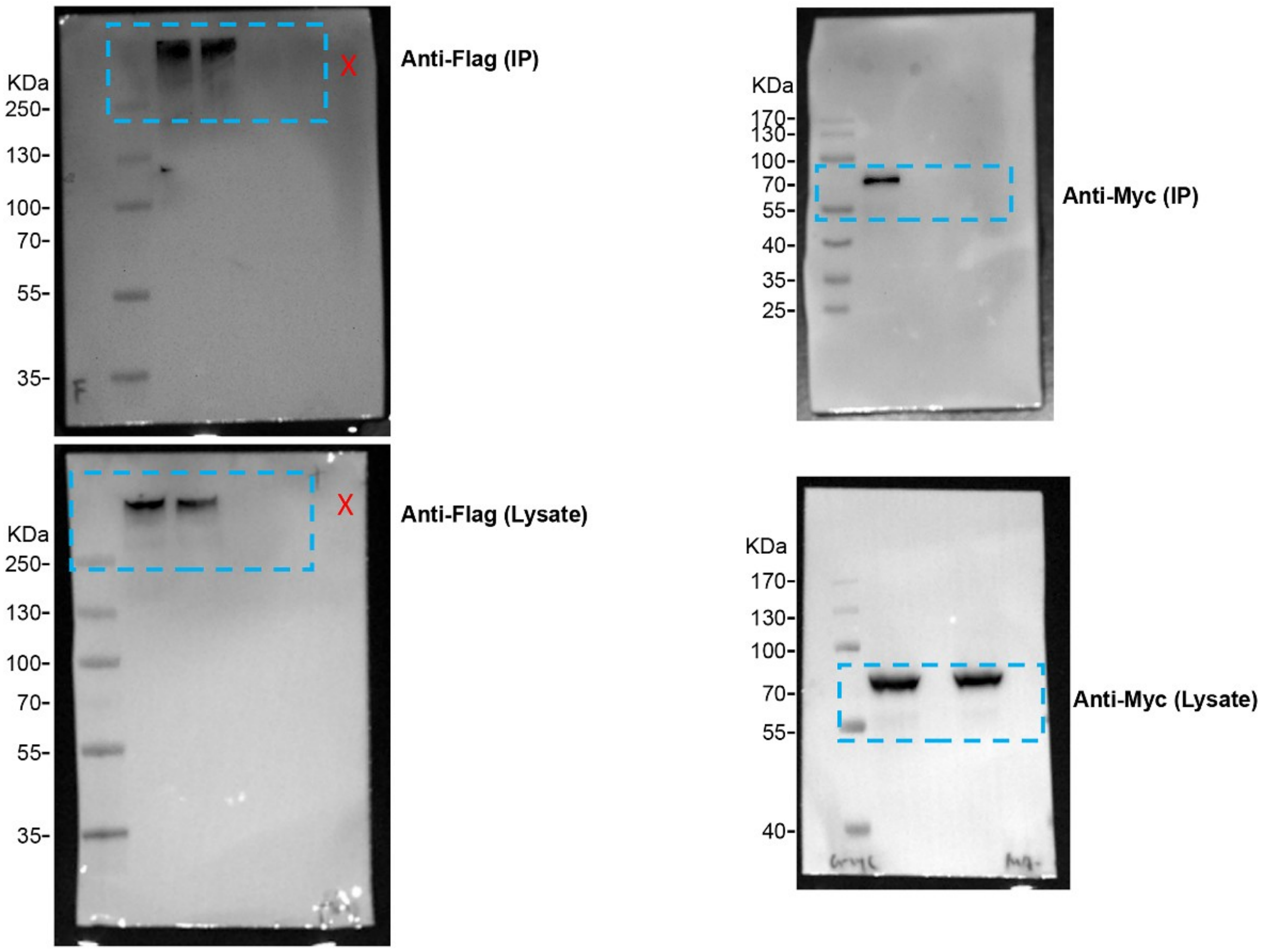

Supplement: Supplementary file 1 [file ppat.1014675.s004.pdf]
